# Dengue Virus: Protection by T Cells, Disease Exacerbation by Antibodies?

**DOI:** 10.1016/j.ebiom.2016.10.031

**Published:** 2016-10-24

**Authors:** Christian Münz

**Affiliations:** Viral Immunobiology, Institute of Experimental Immunology, University of Zürich, Switzerland

The insect transmitted arboviruses among the flaviviruses pose a significant health risk to humans as observed during the recent Zika virus outbreak in South America (Lessler et al., 2016). One of the most prominent members of this group of pathogens is Dengue virus (DENV), which exists in four serotypes, DENV1–4 (Bhatt et al., 2013, Messina et al., 2014). While infection with one serotype confers protection against reinfection with the same serotype and is usually associated with only mild symptoms, exposure to a different serotype can cause severe disease, resulting in vascular leakage, uncontrolled cytokine levels and hemorrhagic fever. Especially delayed exposure to a second serotype from several months to three years after primary DENV infection increases the risk for severe disease (Montoya et al., 2013). This suggests that waning DENV-specific immunity which diminishes protective cross-reactive immune responses against other serotypes can even exacerbate disease. Both the humoral and the cell-mediated arm of the immune system have been suspected to be responsible for this exacerbation. Cross-reactive, but not protective antibody responses might exacerbate infection by antibody-dependent enhancement (ADE) (Zellweger et al., 2010). In addition, original antigenic sin (OAS) has been proposed to focus T cell responses during infection with one serotype towards specificities that upon reinfection with another serotype are not protective, but get re-stimulated to produce immune pathological inflammation (Rothman, 2011). Distinguishing between these two possibilities is of utmost importance to design broadly protective vaccine candidates (Guy et al., 2016).

Supporting the idea that T cell responses may be cross-reactive, the human MHC class I HLA-B*0702 molecule was found to be associated with resistance to severe DENV infection upon exposure to different serotypes (Weiskopf et al., 2013). Building on these findings the study by Elong Ngono and colleagues in this issue of *EBioMedicine* explores CD8^+^ T cell responses against DENV non-structural proteins (NS) 3, 4B and 5 in HLA-B*0702 transgenic type I IFN receptor deficient mice (Elong Ngono et al., 2016). Even though the authors found diminished reactivity of some DENV2 epitope-induced CD8^+^ T cell responses against the respective sequences of the other serotypes, the respective CD8^+^ T cells were able to produce multiple cytokines upon re-stimulation with peptides from all serotypes. More importantly, challenge with DENV2 or DENV3 infection resulted in similar protection after vaccination with DENV2 or DENV1/3/4 derived peptides. These findings suggest that CD8^+^ T cell responses against DENV NS proteins can mediate protective cross-reactive immunity. However, it needs to be determined in the future if this is a particular feature of the protective HLA-B*0702 allele that was used in this study or can be generalized to most HLA haplotypes.

If such protective cross-reactivity can, however, be demonstrated across most HLA haplotypes, then immunodominant CD8^+^ T cell antigens should be preferentially included into DENV-specific vaccination approaches. Following this line of thought, live-attenuated viral vectors, which stimulate CD8^+^ T cell responses most efficiently, might constitute a promising vaccine formulation (Guy et al., 2016). Along these lines, the live-attenuated vaccine of yellow fever virus (YV17D), another arbovirus of the flavivirus family, seems to be among the most successful vaccinations in humans (Gotuzzo et al., 2013). However, the NS antigens of DENV, which seem to provide dominant CD8^+^ T cell stimulation, should be included in such a protective T cell directed vaccination with live-attenuated viruses.

## Conflict of Interest

The author declares no conflict of interest.
